# PREMIM and EMIM: tools for estimation of maternal, imprinting and interaction effects using multinomial modelling

**DOI:** 10.1186/1471-2105-13-149

**Published:** 2012-06-27

**Authors:** Richard Howey, Heather J Cordell

**Affiliations:** 1 Institute of Genetic Medicine, Newcastle University, Central Parkway, Newcastle upon Tyne, NE1 3BZ, UK

**Keywords:** Case/parent trio, Maternal-fetal interaction, Parent-of-origin, Genome-wide association study

## Abstract

**Background:**

Here we present two new computer tools, PREMIM and EMIM, for the estimation of parental and child genetic effects, based on genotype data from a variety of different child-parent configurations. PREMIM allows the extraction of child-parent genotype data from standard-format pedigree data files, while EMIM uses the extracted genotype data to perform subsequent statistical analysis. The use of genotype data from the parents as well as from the child in question allows the estimation of complex genetic effects such as maternal genotype effects, maternal-foetal interactions and parent-of-origin (imprinting) effects. These effects are estimated by EMIM, incorporating chosen assumptions such as Hardy-Weinberg equilibrium or exchangeability of parental matings as required.

**Results:**

In application to simulated data, we show that the inference provided by EMIM is essentially equivalent to that provided by alternative (competing) software packages such as MENDEL and LEM. However, PREMIM and EMIM (used in combination) considerably outperform MENDEL and LEM in terms of speed and ease of execution.

**Conclusions:**

Together, EMIM and PREMIM provide easy-to-use command-line tools for the analysis of pedigree data, giving unbiased estimates of parental and child genotype relative risks.

## Background

Genomewide association studies have popularized the use of the case/control design to detect effects associated with an individual’s own genotype, however many diseases (especially those related to pregnancy outcomes) may in fact be due to more complex effects such as maternal genotype effects, maternal-fetal genotype interactions or parent-of-origin (imprinting) effects. To detect such effects it is necessary to collect genotype data from one or both parents of cases, in addition to genotyping the cases themselves. Two existing popular approaches analyse either genetic data from affected offspring and their mothers (case/mother duos), along with an appropriate control sample [1-3], or else analyse genetic data from affected offspring and both parents (case/parent trios), without use of controls [4-6]. In contrast, our software EMIM uses a multinomial modelling approach [7] that allows the simultaneous consideration of both case/mother duos and/or case/parent trios, with additional child and parent genotype data (such as individual cases and controls, case/father duos and control matings) included when available. The child-parent genotype data can be extracted from standard PLINK-format [8] pedigree files using our companion software PREMIM.

Full details and evaluation of the multinomial modelling approach used by EMIM have been described previously [7]. The early beta version of EMIM described in [7] allowed a more limited set of child-parent configurations than are supported in the current version, and did not include the current full range of optional likelihood assumptions (such as conditioning on parental genotypes (CPG) [6,9]). Most importantly, the companion program PREMIM was not available, limiting the ease with which EMIM could be applied to real data.

### PREMIM: Pedigree file conversion

For each SNP in turn, PREMIM performs a simple algorithm to select from each pedigree the most informative sub-unit of child-parent genotype data. Different pedigree sub-units are chosen in order of preference as listed in Table 1.

**Table 1:** The order of preference of pedigree sub-units chosen by PREMIM for each SNP


**Order**	**Pedigree sub-unit**
1	case/parent trio
2	case/mother duo
3	case/father duo
4	case
5	case parental mating
6	case mother
7	case father
8	control parental mating
9	control/mother duo
10	control/father duo
11	control

There are a number of options that may be given to PREMIM. In particular, it is possible to override the default choice of individuals by stating a proband subject for certain pedigrees. These proband subjects are then chosen as cases (with parents where available). This may be useful to avoid possible bias when larger pedigrees have been ascertained on the basis of a specific affected individual. For larger pedigrees, it is also possible to select multiple case/parent trios or multiple control matings from each pedigree, potentially increasing the power to detect genetic effects. This option does have the potential to generate bias (depending on the analysis options chosen [6,10]), and so results should be interpreted with caution, although we anticipate that most people will apply these types of method to small pedigrees such as child/parent trios, making this issue less of a concern in practice. (Alternative methods for dealing with larger pedigrees, valid under the assumptions of random mating and/or Hardy-Weinberg equilibrium (HWE), have been described by [10,11]).

### EMIM methodology

The basic principle behind EMIM is simple: to test for the existence of (and estimate) genotype relative risk parameters that increase (or decrease) the probability that a child is affected. By default, PREMIM chooses the minor allele to be considered as the ‘risk’ allele, although this option can be overridden if required. We denote by *R*_1_(*R*_2_) the factor by which an individual’s disease risk is multiplied if they possess one (two) risk alleles at a given locus. We denote by *S*_1_(*S*_2_) the factor by which an individual’s disease risk is multiplied if their mother possesses one (two) risk alleles at that locus. We denote by *I*_*m*_(*I*_*p*_) the factor by which an individual’s disease risk is multiplied if they inherit a risk allele from their mother (father). Lastly, to test for mother-child interactions, we denote by *γ*_*ij*_ the factor by which an individual’s disease risk is multiplied if the mother carries *i* risk alleles and the child carries *j* risk alleles. A summary of these relative risk parameters is shown in Table 2. A variety of restrictions may be made on the parameters as desired. For example, a multiplicative model for the effects of the alleles in the mother ($S_{2}=S_{1}^{2}$) or child ($R_{2}=R_{1}^{2}$) may be imposed. In addition, EMIM also supports several alternative previously-proposed paramaterizations for the imprinting and interaction effects [4,5] (see [7] for more details).

**Table 2:** The relative risk parameters estimable by EMIM


**Parameter**	**Description**
*R*_1_	Child has one minor allele (child genotype effect)
*R*_2_	Child has two minor alleles (child genotype effect)
*S*_1_	Mother has one minor allele (maternal genotype effect)
*S*_2_	Mother has two minor alleles (maternal genotype effect)
*γ*_11_	Mother has one minor allele and child has one minor
	allele (mother-child interaction effect)
*γ*_12_	Mother has one minor allele and child has two minor
	alleles (mother-child interaction effect)
*γ*_21_	Mother has two minor alleles and child has one minor
	allele (mother-child interaction effect)
*γ*_22_	Mother has two minor alleles and child has two minor
	alleles (mother-child interaction effect)
*I*_*m*_	The child receives a minor allele from the mother
	(maternally operating imprinting effect)
*I*_*p*_	The child receives a minor allele from the father
	(paternally operating imprinting effect)

As an example, denote the major and minor alleles by 1 and 2, then for a case/parent trio where the genotypes of the mother, father and child are 22, 11, 12, respectively, the penetrance is modelled as:

(1) $$P(\text{child diseased}|g_{m}\;=\;22,g_{f}\;=\;11,g_{c}\;=\;12)\;=\;\alpha R_{1}S_{2}I_{m}\gamma_{21}$$

where *α* is the baseline probability of disease and *g*_*m*_, *g*_*f*_ and *g*_*c*_ are the genotypes of the mother, father and child.

EMIM uses a multinomial model to estimate the relative risk parameters on the basis of observed counts of genotype combinations in case/parent trios as shown in Table 3. EMIM models the 15 different cell probabilities (corresponding to the 15 possible combinations of genotypes that are consistent with Mendelian inheritance) in terms of the desired genotype relative risk parameters (*R*_1_*R*_2_*S*_1_*S*_2_*I*_*m*_*I*_*p*_*γ*_11_*γ*_12_*γ*_21_*γ*_22_). A maximum of 7 parameters are estimable, meaning that not all of these parameters can be estimated simultaneously. Cordell et al. [12] suggested building up models from simpler to more complex via a series of nested hypothesis tests. Given a model for the penetrances in terms of the genotype relative risk parameters, the overall likelihood for the data in Table 3 may be written

(2) $$\overset{15}{\underset{i=1}{\prod }}{\left\{P\left(g_{m_{i}},g_{f_{i}},g_{c_{i}}|\text{child diseased}\right)\right\}}^{n_{i}}$$

where $\left(g_{m_{i}},g_{f_{i}},g_{c_{i}}\right)$ represent the genotypes of a mother, father and child in genotype combination *i*. The probabilities $P(g_{m_{i}},g_{f_{i}},g_{c_{i}}|\text{child diseased})$ may be written in terms of the genotype relative risk parameters of interest and six nuisance parameters *μ*_1_−*μ*_6_(corresponding to mating type stratification parameters as indexed in Table 3, see [4,7,13] for details).

**Table 3:** Observed genotype combinations in case/parent trios


**Genotypes**^**a**^			**Index of**	**Index of CEPG**^**b**^	**Index of CPG**^**c**^	**Observed**
***g***_***m***_	***g***_***f***_	***g***_***c***_	**combination**	**parental mating type**	**parental mating type**	**count**
22	22	22	1	1	1	*n*_1_
22	12	22	2	2	2	*n*_2_
22	12	12	3	2	2	*n*_3_
12	22	22	4	2	3	*n*_4_
12	22	12	5	2	3	*n*_5_
22	11	12	6	3	4	*n*_6_
11	22	12	7	3	5	*n*_7_
12	12	22	8	4	6	*n*_8_
12	12	12	9	4	6	*n*_9_
12	12	11	10	4	6	*n*_10_
12	11	12	11	5	7	*n*_11_
12	11	11	12	5	7	*n*_12_
11	12	12	13	5	8	*n*_13_
11	12	11	14	5	8	*n*_14_
11	11	11	15	6	9	*n*_15_

^a^*g*_*m*_,*g*_*f*_,*g*_*c*_=genotypes of mother, father, child, respectively.

^b^CEPG= conditional on exchangeable parental genotypes.

^c^CPG= conditional on parental genotypes.

If any of the subjects are missing, we no longer have 15 genotype counts as shown in Table 3, but instead we must collapse together rows to express the data in terms of counts of observed genotype combinations. For example, given data for case/mother duos (i.e. all fathers missing), the 7 observable counts are as shown in Table 4. The likelihood for the data in this table may be written

(3) $$\begin{array}{c} \overset{7}{\underset{i=1}{\prod }}{\left\{P(g_{m_{i}},g_{c_{i}}|\text{child diseased})\right\}}^{m_{i}}\\ \;=\overset{7}{\underset{i=1}{\prod }}{\left\{\underset{g_{f}}{\sum }P(g_{m_{i}},g_{f},g_{c_{i}}|\text{child diseased})\right\}}^{m_{i}} \end{array}$$

**Table 4:** Observed genotype combinations in case/mother duos


**Genotypes**^**a**^		**Index of**	**Observed**
*g*_*m*_	*g*_*c*_	combination	count
22	22	1	*m*_1_=*n*_1_ + *n*_2_
22	21	2	*m*_2_=*n*_3_ + *n*_6_
12	22	3	*m*_3_=*n*_4_ + *n*_8_
12	12	4	*m*_4_=*n*_5_ + *n*_9_ + *n*_11_
12	11	5	*m*_5_=*n*_10_ + *n*_12_
11	12	6	*m*_6_=*n*_7_ + *n*_13_
11	11	7	*m*_7_=*n*_14_ + *n*_15_

^a^*g*_*m*_,*g*_*c*_=genotypes of mother, child, respectively.

where $\left(g_{m_{i}},g_{c_{i}}\right)$ represent the genotypes of a mother and child in (Table 4) genotype combination *i*.

In practice, at any given SNP, we observe genotype counts (some of which may equal 0) for the following types of unit: case/parent trios (15 possible genotype combinations); parents of cases (9 possible genotype combinations); case/mother duos (7 possible combinations); case/father duos (7 possible combinations); mothers of cases (3 possible combinations); fathers of cases (3 possible combinations); cases (3 possible combinations). The data for each unit creates a table corresponding to a (possibly collapsed) version of Table 3, and the overall likelihood to be maximized may be constructed as the product of the likelihoods for the individual tables. Similarly, we may add in data for controls (either unaffected individuals or population-based controls of unknown disease status) by further multiplying the likelihood by the product of the likelihoods for a similar set of control tables. EMIM makes use of the following types of control unit: parents of controls (9 possible genotype combinations); control/mother duos (7 possible combinations); control/father duos (7 possible combinations); controls (3 possible combinations). Furthermore, EMIM assumes that the frequencies of the different genotype combinations in control units correspond to those in the general population. This is equivalent to making a rare disease assumption, in the event that the controls are all genuinely unaffected.

By default, EMIM assumes ‘mating symmetry’ [13] (equivalent to a ‘conditional on exchangeable parental genotypes’ (CEPG) [12] model), which corresponds to assuming that parental matings (*g*_*m*_=*ig*_*f*_=*j*) are as likely as matings (*g*_*m*_=*jg*_*f*_=*i*). This results in the estimation of six mating type stratification parameters [13]*μ*_1_−*μ*_6_(see Table 3). Two more restricted (and therefore potentially more powerful) models are also available in EMIM: 

1. A model that assumes parental allelic exchangeability (PAE) [2] (which corresponds in this context to assuming that *μ*_4_=*μ*_3_)

2. A model that assumes Hardy-Weinberg equilibrium (HWE) and random mating, estimating a single allele frequency parameter in place of the six mating type stratification parameters.

In addition to these more restricted models, a less restricted ‘conditional on parental genotypes’ (CPG) [2,9,12] model (that results in the estimation of nine mating type stratification parameters *μ*_1_−*μ*_9_, see Table 3) is also available. This model would be expected to be less powerful than the CEPG, PAE or HWE models, but should be more robust to any departure from mating symmetry, PAE or HWE.

EMIM reads in genotype data from input files created by PREMIM. In addition, there are two other files required by EMIM. Firstly, a file ‘emimmarkers.dat’, which provides the minor allele frequencies for each SNP (used as starting values in the maximization algorithm). These can optionally be estimated by PREMIM using the pedigree data, although other (e.g. population-based) sources for this information may be preferred where available. (See [7] for an investigation of EMIM’s sensitivity to misspecification of the assumed or estimated allele frequencies). The other required file is a parameter file ‘emimparams.dat’, describing the type of analysis that EMIM should perform, which parameters to estimate, and which assumptions (such as HWE or PAE) should be made.

## Implementation

PREMIM is written in C++ and for a binary pedigree file with 913 pedigrees, 1730 subjects and 45323 SNPs it takes 19 seconds to process on a Six-Core AMD Opteron^TM^ Processor with 2.6 GHz CPUs. EMIM is written in FORTRAN 77 and makes use of a subroutine MAXFUN, originally written as part of the S.A.G.E. [14] package. For these same data (pre-processed by PREMIM) on the same machine, EMIM takes 1 minute and 22 seconds to perform an analysis to test for multiplicative child genotype effects, assuming HWE. For larger data sets, EMIM and PREMIM have options that allow easy parallel processing by dividing the SNPs to analyse into different batches.

## Results and discussion

### Example analysis using simulated data

**Figure 1:**
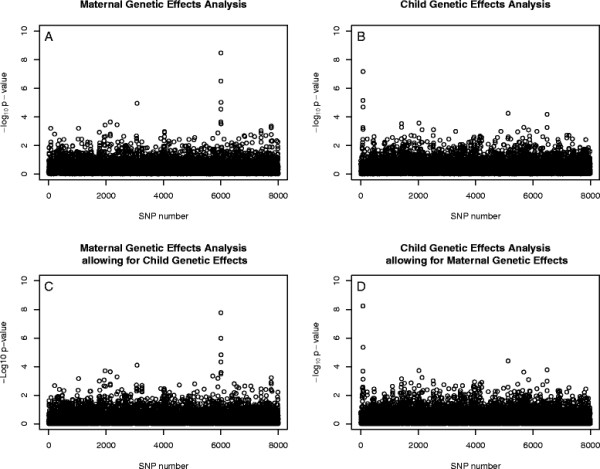
**Genetic Effects.** Plot of the $-\underset{10}{\log}$ p-values for each SNP given by EMIM to detect: (**A**) child genetic effects; (**B**) maternal genetic effects; (**C**) maternal genetic effects whilst allowing for child effects; and (**D**) child genetic effects whilst allowing for maternal effects.

We used the program SimPed [15] to generate a single replicate of simulated data for 200 case/parent trios, 200 case/mother duos, 200 control/mother duos and 1000 unrelated controls at 8000 SNPs across a chromosome. We used a simplified linkage disequilibrium (LD) model that assumed LD operated in haplotype blocks, each of length 8 SNPs. We simulated child genotype effects (*R*_1_=1.5 and *R*_2_=2.25) at SNP 76 and maternal genotype effects (*S*_1_=2 and *S*_2_=3) at SNP 6004. We then used EMIM to test for maternal effects, with and without allowing for child genotype effects (Figure 1C, Figure 1A), and to test for child genotype effects, with and without allowing for maternal effects (Figure 1D, Figure 1B). In all four analyses, we see a strong signal at the correct location, with the high significance probably due to the relatively large effect sizes assumed.

A tutorial for this example (with a listing of the required commands) is available on the PREMIM and EMIM website: http://www.staﬀ.ncl.ac.uk/richard.howey/emim/example.html

### Comparison of HWE, PAE, CEPG and CPG likelihoods

**Figure 2:**
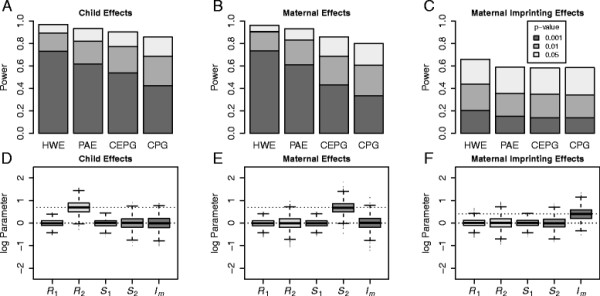
**Comparison of Likelihood Assumptions in EMIM.** Results from simulated data. (**A-C**) The power of likelihood ratio tests to achieve significance levels (p-values) of 0.001, 0.01 and 0.05 for different simulated effects and likelihood assumptions: HWE - Hardy-Weinberg Equilibrium; CEPG - Conditional on Exchangeable Parental Genotypes; CPG - Conditional on Parental Genotypes; PAE - Parental Allelic Exchangeability. (**D-F**) Box plots on a log-scale of parameter estimates for *R*_1_, *R*_2_, *S*_1_, *S*_2_and *I*_*m*_, assuming HWE. Dotted lines show the true parameter values.

The power to detect genetic effects can vary depending on the assumptions made. As a demonstration, we simulated 1000 replicates of data at a single SNP for a sample consisting of 50 of each of the following units: case/parent trios, case/mother duos, case/father duos, control matings, control/mother duos and control/father duos. We assumed either a child genotype effect (*R*_2_=2), a maternal genotype effect (*S*_2_=2), or a maternal imprinting effect (*I*_*m*_=1.8). PREMIM and EMIM were used to estimate the parameters *R*_1_, *R*_2_, *S*_1_, *S*_2_ and *I*_*m*_ for each different likelihood assumption and for each set of simulated data. Figure 2(A-C) shows that the power to detect the relevant effect decreases as one makes less restrictive (but potentially more robust) assumptions, while Figure 2(D-E) shows that unbiased parameter estimation is achieved using the most restrictive assumption (HWE) (provided that assumption is correct). Similar unbiased parameter estimation is achieved for the other likelihood assumptions, when they are met (data not shown).

### Effect of missing data on power

**Figure 3:**
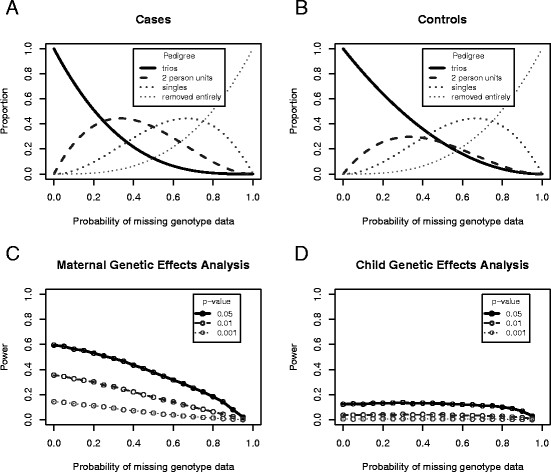
**Effect of Missing Genotype Data.** Plots showing the effect as the probability of missing genotype data is increased for data simulated with a maternal genetic effect. **A**: The expected proportions of different types of pedigree unit output by PREMIM from a set of case/parent trios, for different probabilities of missing genotypes. **B**: The expected proportions of different types of pedigree unit output by PREMIM from a set of control trios, for different probabilities of missing genotypes. **C**: Power of EMIM to detect maternal genetic effects (by estimating parameters *S*_1_and *S*_2_). **D**: Power of EMIM to detect maternal genetic effects masquerading as child genetic effects (by estimating parameters *R*_1_and *R*_2_).

As a demonstration of the effect that missing data has on the power, we performed analyses at a single SNP using simulated data (10,000 replicates, each replicate consisting of 100 case/parent trios and 100 control/parent trios) and assuming a range of probabilities of missing genotype data. We assumed a maternal genotype effect (*S*_1_=1.5, *S*_2_=2.25). The expected proportion of pedigree units of different types remaining in the analysis are shown in Figure 3A and Figure 3B respectively. The trios are all present when there is no missing data, but the expected proportion quickly decreases when the probability of missing genotype data is increased. The expected proportion of the other pedigree types then increases, but subsequently decreases and converges to 0 as the probability of missing data approaches 1. The power to detect the maternal genetic effects (when correctly modelled) also decreases with increasing proportion of missing data (Figure 3C). An advantage of the EMIM framework is that it makes efficient use of data from all possible available individuals, allowing one to recover information even from incompletely genotyped trios.

Buyske [16] pointed out that maternal genotype effects can masquerade as child genotype effects, if analysed as such. If the maternal genetic effects are incorrectly modelled as child genetic effects (Figure 3D), we find limited power to detect these effects even when there is no missing data. Increasing the proportion of missing data has little effect on the power of this analysis, until the probability of missing genotype data becomes very large (e.g. more than 80%).

### Comparison with MENDEL

Several other software packages exist that allow testing and estimation of genotype relative risk parameters similar to those tested in EMIM. One such package is MENDEL [17]. MENDEL most easily allows the estimation and testing of mother-child interaction effects via the maternal-fetal genotype incompatibility (MFG) test [5], although a “Generalized Risk” analysis that allows implementation of more complex user-defined paramaterizations (through the imposition of various parameter restrictions) is also available.

We used computer simulations (500 replicates each with 200 case parent trios) to compare the performance of MENDEL and EMIM under three different comparable models: 

1. **Model 1**. This model has been used to test for RhD incompatibility [18] and estimates the relative risk corresponding to the mother having no risk alleles and the child one risk allele. MENDEL was used to estimate this one relative risk parameter by setting the sex-specific effects (parameters MFG_M and MFG_F in MENDEL) to be equal. The equivalent single parameter *γ*_01_(corresponding to the parametrization of [5,18]) was estimated in EMIM. The data were simulated assuming *γ*_01_=2.

2. **Model 2**. This model has been used to test for non-inherited maternal antigens (NIMA) on rheumatoid arthritis (RA) [19] and consists of three parameters (ignoring sex-specific MFG testing): a relative risk parameter (*γ*_10_) for MFG when the mother has one risk allele and the child has no risk alleles, and two parameters for child effects when the child has one or two risk alleles. In order to compare EMIM with MENDEL under this model, we used PREMIM to reassign which allele should be considered as the risk allele by EMIM. A model equivalent to MENDEL’s NIMA model can then be fit in EMIM by estimating parameters (with respect to the reassigned allele) *R*_1_*R*_2_and *γ*_12_. Data were simulated assuming an MFG effect *γ*_10_=2. The power to detect the the MFG effect in either MENDEL or EMIM was calculated by considering twice the difference between the negative log likelihood from a model that includes all three parameters (*R*_1_*R*_2_and the MFG parameter) and that from a model where the MFG parameter has been removed.

3. **Model 3.** This MENDEL model is a general MFG test consisting of one relative risk parameter for each of the 7 mother/child genotype combinations. The relative risk parameter denoted U_00 in the MENDEL documentation (corresponding to the situation where the mother and child have no risk alleles) was set to 1 and not estimated to avoid over-parametrization. The other 6 parameters, U_22, U_21, U_12, U_11, U_10, U_01, were estimated. The 6 parameters estimated by EMIM were *R*_1_, *R*_2_, *S*_1_, *S*_2_, *γ*_11_and *γ*_22_. These parameters are not indvidually equivalent to the 6 MENDEL parameters, but the models as a whole can be shown to be equivalent. Data for this comparison were simulated assuming *R*_1_=*S*_1_=*γ*_11_=*γ*_22_=1.5 and *R*_2_=*S*_2_=2.25.

**Figure 4:**
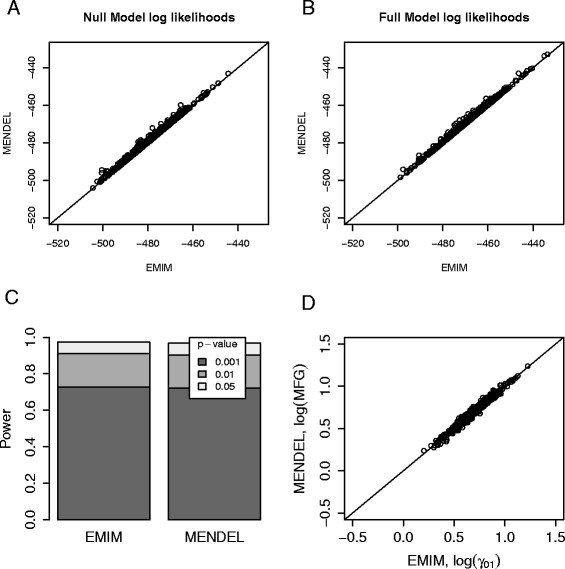
**Mother-Child Interaction Effects Comparison with MENDEL, RHD.** Plots showing the comparison of EMIM and MENDEL - “option 26, Model 1: RHD” using simulated data. **A**: Plot of the null model log likelihood values calculated using EMIM and MENDEL. **B**: Plot of the full (alternative) log likelihood values calculated using EMIM and MENDEL. **C**: The power to detect a genetic effect for p-values of 0.05, 0.01 and 0.001. **D**: Plot of the MFG parameter estimates calculated using EMIM and MENDEL.

**Figure 5:**
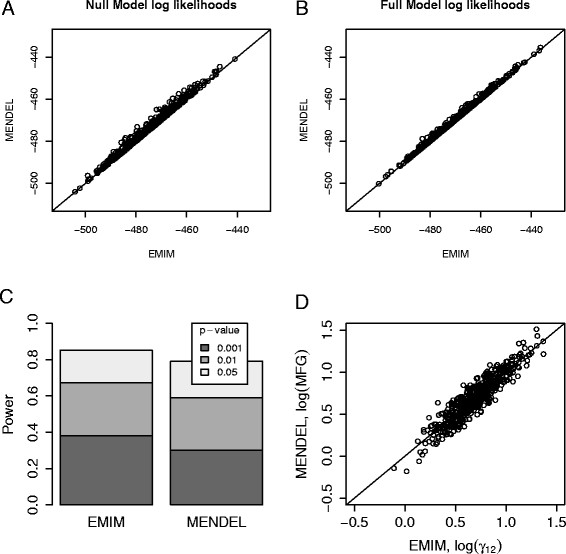
**Mother-Child Interaction Effects Comparison with MENDEL, NIMA.** Plots showing the comparison of EMIM and MENDEL - “option 26, Model 2: NIMA” using simulated data. **A**: Plot of the null model log likelihood values (with child effects fitted, but no MFG interaction effect) calculated using EMIM and MENDEL. **B**: Plot of the full (alternative) log likelihood values (fitting both child effects and MFG effect) calculated using EMIM and MENDEL. **C**: The power to detect the MFG effect for p-values of 0.05, 0.01 and 0.001. **D**: Plot of the MFG parameter estimates calculated using EMIM and MENDEL.

Figures 4 and 5 show a comparison of the null model (no estimated parameters) and the full model log likelihoods from EMIM and MENDEL, for Models 1 and 2 respectively. EMIM was set to assume HWE (since MENDEL assumes HWE by default). We see that the null and full model log likelihoods from the two programs are are very similar (Figures 4(A), 4(B), 5(A), 5(B)), resulting in approximately equal powers and parameter estimates (Figures 4(C), 4(D), 5(C), 5(D)). For Model 3, EMIM and MENDEL similarly gave approximately equal log likelihoods and powers (results not shown).

One difference between EMIM and MENDEL was the time taken to perform the analysis, with EMIM performing considerably quicker than MENDEL. For example, the time to run model 3 (with 200 case/parent trios) showed that PREMIM and EMIM combined took 0.0257 seconds and MENDEL took 6.45 seconds (averaged over 300 runs). This shows that PREMIM and EMIM combined were approximately 250 times faster than MENDEL in this example. The same analysis with 400 case/parent trios gave times of 0.0302 seconds for PREMIM and EMIM combined and 14.3 seconds for MENDEL (averaged over 300 runs), showing PREMIM and EMIM to be approximately 472 times faster then MENDEL. A possible reason for the difference in running times is the fact that the extended MFG model [11] implemented in MENDEL is a slightly more complicated model than the parent/offspring trio model implemented in EMIM (thus providing MENDEL with the ability to analyse larger pedigrees).

### Comparison with LEM

Another program with the capability to analyse complex genetic effects (most notably mother/child/imprinting effects) is LEM [20]. LEM is a Windows-based log-linear modelling program designed primarily to be used via a graphical user interface, although it is possible to run it from the DOS command line, in order to implement scripts that allow the analysis of large numbers of loci or replicates. LEM takes an input parameter file which defines the model, the parameters to be estimated and the name of the input data file. We created input parameter and data files based on examples provided by the authors of LEM [20] for case/parent trios and by [21] for case/mother and control/mother duos. 

1. **Case/parent trios.** SimPed [15] was used to simulate a single replicate of data at 8000 SNPs across a chromosome for 4000 case/parent trios. Child effects (*R*_1_=1.5, *R*_2_=2.25) were simulated at SNP number 1004 and maternal effects (*S*_1_=2, *S*_2_=3) were simulated at SNP number 6004. In both EMIM and LEM we tested for maternal effects while allowing for child and maternal imprinting effects (i.e. we compared an alternative 5-parameter model (*R*_1_*R*_2_*S*_1_*S*_2_*I*_*m*_) with a null 3-parameter model (*R*_1_*R*_2_*I*_*m*_)). We calculated the p-value for LEM on the basis of the reported log likelihoods by using the Wald statistic as a ^*χ*2^value with 2 degrees of freedom. (The p-value reported by LEM was not suitable as it is only given to 3 decimal places, which was insufficient for SNPs with p-values less than 1^0−3^).

2. **Case/mother duos and control/mother duos.** Again, data were simulated at 8000 SNPs but this time for 2000 case/mother duos and 2000 control/mother duos. Child effects (*R*_1_=1.5, *R*_2_=2.25) were simulated at SNP number 1000 and maternal effects (*S*_1_=2, *S*_2_=3) were simulated at SNP number 6004. In both EMIM and LEM we tested for maternal and child effects i.e. we compared a null model with no fitted parameters to an alternative model with parameters (*R*_1_, *R*_2_, *S*_1_, *S*_2_).

**Figure 6:**
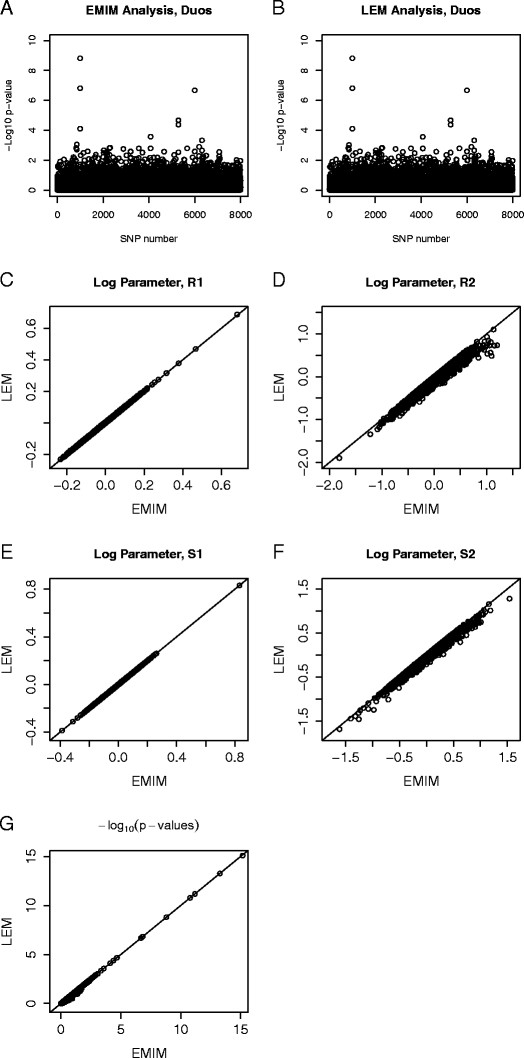
**Comparison of EMIM and LEM for Child/Mother Duos.** Plots showing the comparison of EMIM and LEM using simulated data for 2000 case/mother duos and 2000 control/mother duos, assuming *R*_1_=1.5 and *R*_2_=2.25 at SNP number 1000 and *S*_1_=2 and *S*_2_=3 at SNP number 6004. Plots of the $-\underset{10}{\log}$ p-values for each SNP to detect child and maternal effects by: **A**: EMIM and **B**: LEM. Plots of the log parameters values for: **C**: *R*_1_; **D**: *R*_2_; **E**: *S*_1_and **F**: *S*_2_. **G**: Plot of the $-\underset{10}{\log}$ p-values for the alternative versus the null model calculated using EMIM and MENDEL.

A comparison of EMIM versus LEM for the case/mother and control/mother duos is shown in Figure 6. Figure 6(A) and 6(B) show that the p-values across the chromosome appear to be indistinguishable, and Figure 6(G) shows that the p-values for each SNP from the two programs are indeed approximately equal. Figures 6(C) and 6(E) show that the estimates of *R*_1_ and *S*_1_ are approximately equal and Figures 6(D) and 6(F) show that *R*_2_and *S*_2_ are also approximately equal, but with more variability.

**Figure 7:**
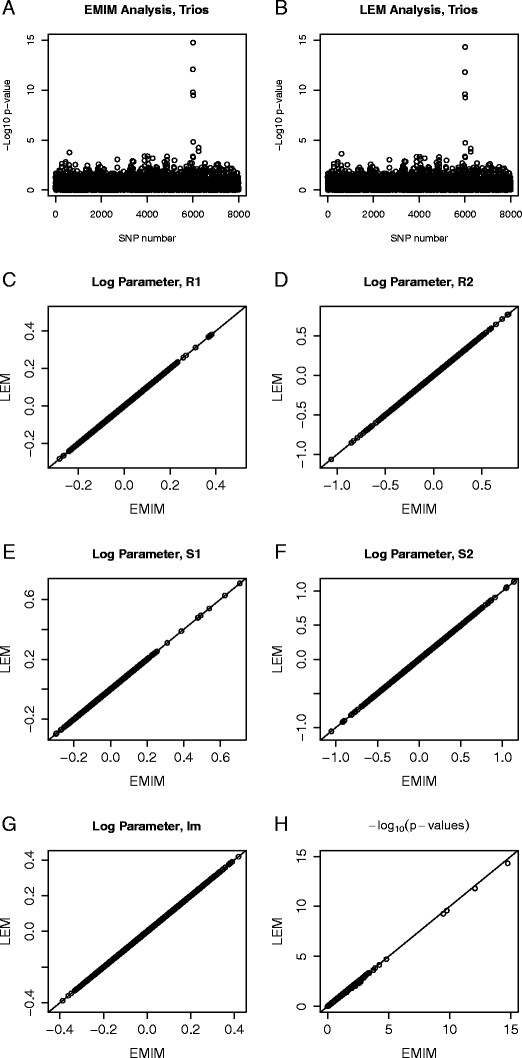
**Comparison of EMIM and LEM for Case/Parent Trios.** Plots showing the comparison of EMIM and LEM using simulated data for 4000 case/parent trios, assuming *R*_1_=1.5 and *R*_2_=2.25 at SNP number 1004 and *S*_1_=2 and *S*_2_=3 at SNP number 6004. Plots of the $-\underset{10}{\log}$ p-values for each SNP to detect maternal effects given child and imprinting effects by: **A**: EMIM and **B**: LEM. Plots of the log parameters values for: **C**: *R*_1_; **D**: *R*_2_; **E**: *S*_1_; **F**: *S*_2_; **G**: *I*_*m*_. **H**: Plot of the $-\underset{10}{\log}$ p-values for the alternative (5-parameter) model versus the null (3-parameter) model calculated using EMIM and MENDEL.

Figure 7 shows the same plots for the case/parent trios, but with the addition of estimates for the extra parameter *I*_*m*_. We see that the p-values and parameter estimates provided by the two programs are virtually indistinguishable.

These results indicate that the inference provided by LEM and EMIM is essentially identical. This is as expected given the mathematical equivalence [7,22] between the multinomial model fit by EMIM and the log linear model fit by LEM. The main difference between the programs is the time taken to perform the analysis, with EMIM performing considerably quicker than LEM. For example, the time taken to run the case/mother and control/mother duos analysis across 8000 SNPs in PREMIM/EMIM was 1 minute 21 seconds on a Linux machine (6-Core AMD Opteron^TM^ Processor with 2.6 GHz CPUs) or 2 minutes 4 seconds on Windows (using a 2-core Intel^TM^ Processor with 2.93 GHz CPUs), whereas the same analysis in LEM took 16 hours, 52 minutes and 8 seconds on Windows (via the DOS command line). The difference in speed between the two programs for the case/parent trios analysis was not as extreme, with PREMIM/EMIM taking 3 minutes 7 seconds on Linux or 4 minutes 49 seconds on Windows, versus LEM’s time of 63 minutes 58 seconds on Windows. The improved speed for the LEM trios analysis was most likely due to the fact that it took fewer steps than the duos analysis during the likelihood maximization process (possibly on account of the fact that the example parameter file we were using requested the program to switch to using a Newton-Raphson algorithm following 10 iterations of an EM algorithm). It is possible that differences between maximization algorithms and convergence criteria could account for some of the differences in speed between PREMIM/EMIM and LEM; we found it difficult to determine how to obtain precise control over such factors in LEM and were forced to use input files that very closely matched the examples provided by [20,21]. Another factor influencing speed could be the fact that LEM does not (as far as we are aware) allow the input of multiple SNPs simultaneously, meaning that we had to create and read into LEM a separate input file for each SNP analysed.

## Conclusions

Here we have presented two new computer tools, PREMIM and EMIM, for the estimation of parental and child genetic effects, based on genotype data from a variety of different child-parent configurations. The current version of EMIM improves upon the early beta version described in [7] by allowing a larger set of possible child-parent configurations, a larger range of optional likelihood assumptions, and by the development of the companion program, PREMIM, for generating the required input files from standard PLINK-format files, considerably improving the ease with which EMIM can be applied to real data.

In application to simulated data, we have shown that the inference provided by EMIM is essentially equivalent to that provided by alternative (competing) software packages such as MENDEL and LEM. EMIM does have the advantage of allowing easy implementation of a wider class of models than are most easily implemented in MENDEL and LEM, although the expert MENDEL/LEM user could probably achieve the same model flexibility through judicious choice of parameter restrictions. However, PREMIM and EMIM (used in combination) considerably outperform MENDEL and LEM in terms of speed of execution, an advantage that is likely to be all the more important when applying these approaches to large-scale data sets such as those generated in genome-wide association studies. To allow further increases in speed, PREMIM and EMIM also have the advantage of allowing easy parallel processing (e.g. on a computer cluster) by dividing the SNPs to analyse into different batches.

Limitations of PREMIM and EMIM include the fact that larger pedigrees are divided into case/parent or control/parent trios (or smaller sub-units) prior to analysis, and the fact that SNPs are analysed one at a time, without borrowing information from neighbouring markers (e.g. on the basis of regional linkage disequilibrium patterns). Methods for dealing with larger pedigrees, valid under the assumptions of random mating and/or Hardy-Weinberg equilibrium (HWE), have been described by [10,11], while [23] present an approach that models haplotypes rather than individual SNPs, allowing the borrowing of information (including information on parent-of-origin or missing genotype data) across neighbouring SNPs. Both of these features would be valuable additions to future releases of our software. Nevertheless, the current versions of EMIM and PREMIM provide easy-to-use command-line tools for the analysis of pedigree data, allowing testing and estimation of a variety of parental and child genotype relative risks.

## Availability and requirements

**Project name:** EMIM and PREMIM **Project home page:**http://www.staﬀ.ncl.ac.uk/richard.howey/emim/**Operating systems:** Windows and Linux executables; FORTRAN and C++ source code **Programming language:** FORTRAN and C++ **Other requirements:** None **Licence**: GNU General Public License **Any restrictions to use by non-academics:** None

## Competing interests

The authors declare that they have no competing interests.

## Authors’ contributions

RH developed the PREMIM software, performed computer simulations and drafted the manuscript. HJC conceived the experiment, developed the EMIM software and revised the manuscript. Both authors read and approved the final manuscript.

## Author’s information

HJC is Professor of Statistical Genetics and a Wellcome Senior Fellow at the Institute of Genetic Medicine, Newcastle University, UK. RH is a Research Associate at the Institute of Genetic Medicine, Newcastle University, UK.
